# Nanoformulated herbal compounds: enhanced antibacterial efficacy of camphor and thymol-loaded nanogels

**DOI:** 10.1186/s12906-024-04435-z

**Published:** 2024-04-02

**Authors:** Abbas Abdollahi, Narges Fereydouni, Hamid Moradi, Abolfazl Karimivaselabadi, Elham Zarenezhad, Mahmoud Osanloo

**Affiliations:** 1 Department of Microbiology, School of Medicine, Fasa University of Medical Sciences, Fasa, Iran; 2 Noncommunicable Disease Research Center, Fasa University of Medical Sciences, Fasa, Iran; 3 Student Research Committee, Fasa University of Medical Sciences, Fasa, Iran; 4 Department of Clinical Biochemistry, School of Medicine, Fasa University of Medical Sciences, Fasa, Iran; 5 Department of Clinical Biochemistry, School of Medicine, Shiraz University of Medical Sciences, Shiraz, Iran; 6 Department of Medical Nanotechnology, School of Advanced Technologies in Medicine, Fasa University of Medical Sciences, Fasa, Iran

**Keywords:** Camphor, Thymol, Nanogel, Antibacterial properties, Nanotechnology

## Abstract

Herbal components are highly useful assets for the advancement of novel antibacterial drugs. Nanotechnology holds great promise as an approach to enhance the effectiveness and develop the composition of these substances. The study developed nanogels incorporating camphor, thymol, and a combination derived from the initial nanoemulsions with particle sizes of 103, 85, and 135 nm, respectively. The viscosity of nanogels and the successful loading of compounds in them were examined by viscometery and ATR-FTIR studies. The bactericidal properties of the nanogels were examined against four bacterial strains. The nanogel containing camphor and thymol at 1250 µg/mL concentration exhibited complete growth suppression against *Pseudomonas aeruginosa* and *Staphylococcus aureus*. The thymol nanogel at 1250 µg/mL and the camphor nanogel at 2500 µg/mL exhibited complete inhibition of growth on *Listeria monocytogenes* and *Escherichia coli*, respectively. Both nanogels showed favorable effectiveness as antibacterial agents and could potentially examine a wide range of pathogens and in vivo studies.

## Introduction

Bacterial infections pose significant difficulties to global healthcare systems, and the misuse of antibiotics has led to bacterial resistance [1, 2]. *Staphylococcus aureus* and *Listeria monocytogenes* are two types of bacteria classified as gram-positive. These bacteria cause significant health risks and provide various obstacles. *S. aureus* is responsible for skin and soft tissue infections, bacteremia, osteomyelitis, endocarditis, and pneumonia [3, 4]. *L. monocytogenes* is a type of bacteria that can lead to many health problems, such as listeriosis, gastroenteritis, septicemia, and issues in the central nervous system [5]. Furthermore, *Pseudomonas aeruginosa* and *Escherichia coli* are two types of bacteria that belong to the gram-negative category and can contribute to severe diseases in humans. *P. aeruginosa* is an opportunistic organism that causes nosocomial infections, such as wound infections, burn skin, otitis, keratitis, urinary tract infections, and ventilator-associated pneumonia [6, 7]. In addition, *E. coli* can be a pathogenic agent for various gastrointestinal and non-gastrointestinal diseases, including diarrhea, bacteremia, cystitis, and abdominal infections [8].

Herbal-derived substances, including essential oils, extractions, or their main constituents, are valuable resources for developing novel antibacterial agents [9–11]. Camphor is a white crystalline solid material with a strong aroma usually obtained from the bark of the *Cinnamomum camphora* tree through distilling [12]. Camphor exhibits potent antibacterial properties against various bacteria, including *Streptococcus mutants*, *Enterococcus faecalis*, and *S. aureus* [13, 14]. This terpenoid has been used as an antiseptic, culinary spice, cold treatment, and aphrodisiac [15, 16]. Due to its significant hydrocarbon moiety, camphor demonstrates a nonpolar nature; it does not readily dissolve in water [17].

Thymol is another herbal terpenoid that is derived from many essential oils, particularly thyme spp. It has various biological effects, including flavoring, antioxidant, anti-inflammatory, local anesthetic, antinociceptive, antiseptic, and antifungal effects [18, 19]. However, it is renowned for its formidable antibacterial attributes against a wide range of pathogens, such as *S. aureus* and *E. coli* [20, 21].

The solubility of most herbal active agents, such as camphor and thymol, in water is low [22, 23]. On the other hand, it is necessary to enhance their solubility to utilize them as disinfectants. Incorporating herbal compounds in nanoformulations has recently been introduced to enhance their effectiveness by augmenting solubility [24, 25]. Nanogels with three-dimensional networks are widely used in developing topical drug delivery systems due to their ability to effectively include both hydrophilic and hydrophobic pharmaceuticals [26, 27]. In addition, nanogels exhibit considerable biocompatibility, biodegradability, sufficient stability, and superior drug-loading capabilities than other nanocarriers [28, 29].

For the first time, nanogel containing camphor was prepared. A comprehensive comparison was then made between its antibacterial effects with nanogel containing thymol and thymol-camphor containing nanogel. Our study focuses on investigating the antibacterial effects of these nanogels on four different types of bacteria, including *P. aeruginosa*, *E. coli*, *S. aureus*, and *L. monocytogenes*.

## Materials and methods

*Pseudomonas aeruginosa* (ATCC 27,853), *Escherichia coli* (ATCC 25,922), *Staphylococcus aureus* (ATCC 25,923), *and Listeria monocytogenes* (ATCC 7644) were acquired from the Pasteur Institute of Iran. Camphor, thymol, tween 80, Muler hinton broth, and Muler hinton agar were purchased from Merck Chemicals (Germany). Carboxymethylcellulose (CMC) was purchased from Sigma-Aldrich (USA).

### Preparation and characterizations of nanogels

The nanogel containing camphor, thymol, and a combination of camphor-thymol was prepared using their primary nanoemulsion, as outlined in our earlier research [30]. In summary, camphor, thymol (either separately or in combination), ethanol, and tween 80 were initially mixed. Then distilled water was gradually added until the total volume reached 5000 µL (while stirring at a speed of 2000 rpm for a duration of 60 min at room temperature). The particle size and particle size distribution (SPAN) of the nanoemulsions were examined using a DLS-type instrument (K-One-Nano-ltd). The SPAN values were determined using the formula d90-d10/d50, where d represents the diameter and 10, 50, and 90 correspond to the percentiles of particles with diameters lower than these thresholds.

The prepared nanoemulsions containing camphor, thymol, and camphor-thymol were gelified by adding CMC, the thickening agent. The gelation process was accomplished by agitating for 15 h at a speed of 2000 rpm; the prepared nanogels were named C-NGEL, T-NGEL, and CT-NGEL. In addition, a gel blank (Gel(-C)) was developed using similar procedures but without including camphor or thymol. Table 1 provides an in-depth description of the created nanogels and their constituents.

The rheological properties of the nanogels were examined using a rheometer apparatus (MCR-302- Anton Paar- Austria) to determine their viscosity under various shear rates. In addition, the incorporation of camphor and thymol into nanogels was examined using Attenuated Total Reflectance-Fourier-Transform Infrared (ATR-FTIR) spectroscopy (Tensor II, Bruker, Germany). The spectra of camphor, thymol, CMC, Gel(-C), C-NGEL, T-NGEL, and CT-NGEL were measured in the 400–4000 cm^− 1^ range. Furthermore, the stability of the nanogels was assessed by storing them at ambient temperature and in a refrigerator for a duration of 6 months, followed by a visual examination.

**Table 1:** Ingredients used in the nanogels preparation

Samples	Ingredients				
	camphor	thymol	ethanol	tween 80	CMC
C-NGEL	2% w/v	-	0.5% w/v	6% w/v	3.5% w/v
T-NGEL	-	2% w/v	0.5% w/v	4% w/v	3.5% w/v
CT-NGEL	1% w/v	1% w/v	0.5% w/v	8% w/v	3.5% w/v
Gel(-C)	-	-	0.5% w/v	8% w/v	3.5% w/v

**Fig. 1:**
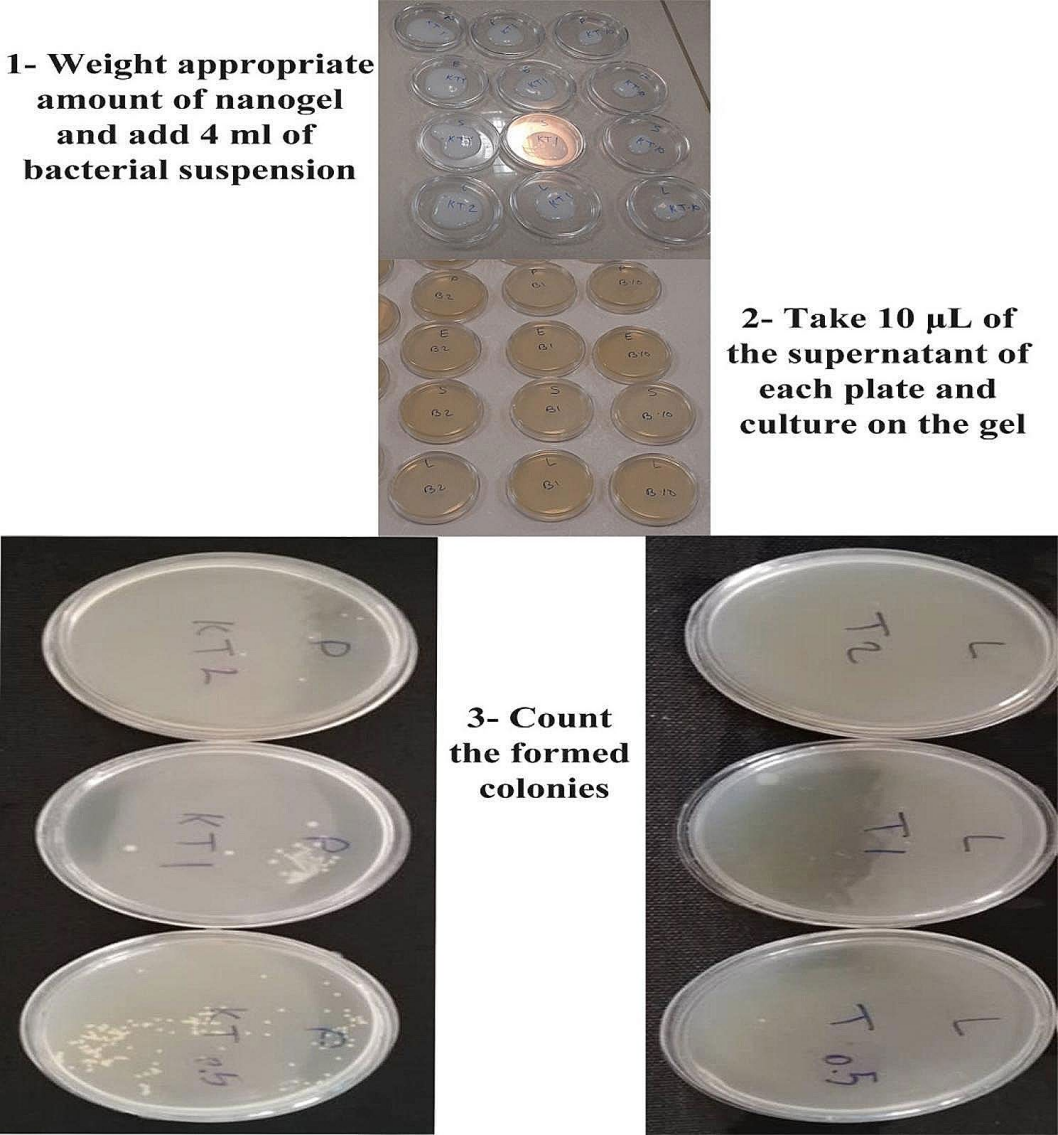
Steps for conducting antibacterial testing

The antimicrobial properties of the samples were examined using the ATCC100 methodology outlined in our prior publication [31]. The steps for conducting antibacterial tests are shown in Fig. 1. In summary, 1, 0.5, and 0.25 g of C-NGEL, T-NGEL, and CT-NGEL were individually applied to 5-centimeter plates. Subsequently, four milliliters of each bacterial suspension, with a concentration of 2 × 10^5^ CFU/mL, were added. As the nanogels contained 2% w/v (20.000 µg/mL) of active agent, the concentration of samples was fixed at 5000, 2500, and 2500 µg/mL. In addition, 1 gram of Gel(-C) was introduced to three plates as the negative control, while the control plates were left untreated. Subsequently, the plates were placed in a shaking incubator at a temperature of 37 °C for 24 h. Afterward, 10 µL of plate suspensions were cultivated on muller hinton gel and incubated for 24 h. The quantity of colonies cultivated on agar gels was enumerated and evaluated with the control group. The growth inhibition was determined by calculating the difference between the number of colony-forming units (CFUs) in the control group and the sample group, divided by the number of CFUs in the control group, and then multiplied by 100 (CFU control − CFU sample /CFU control ×100).

## Results

### Physicochemical properties of the nanogels

**Fig. 2:**
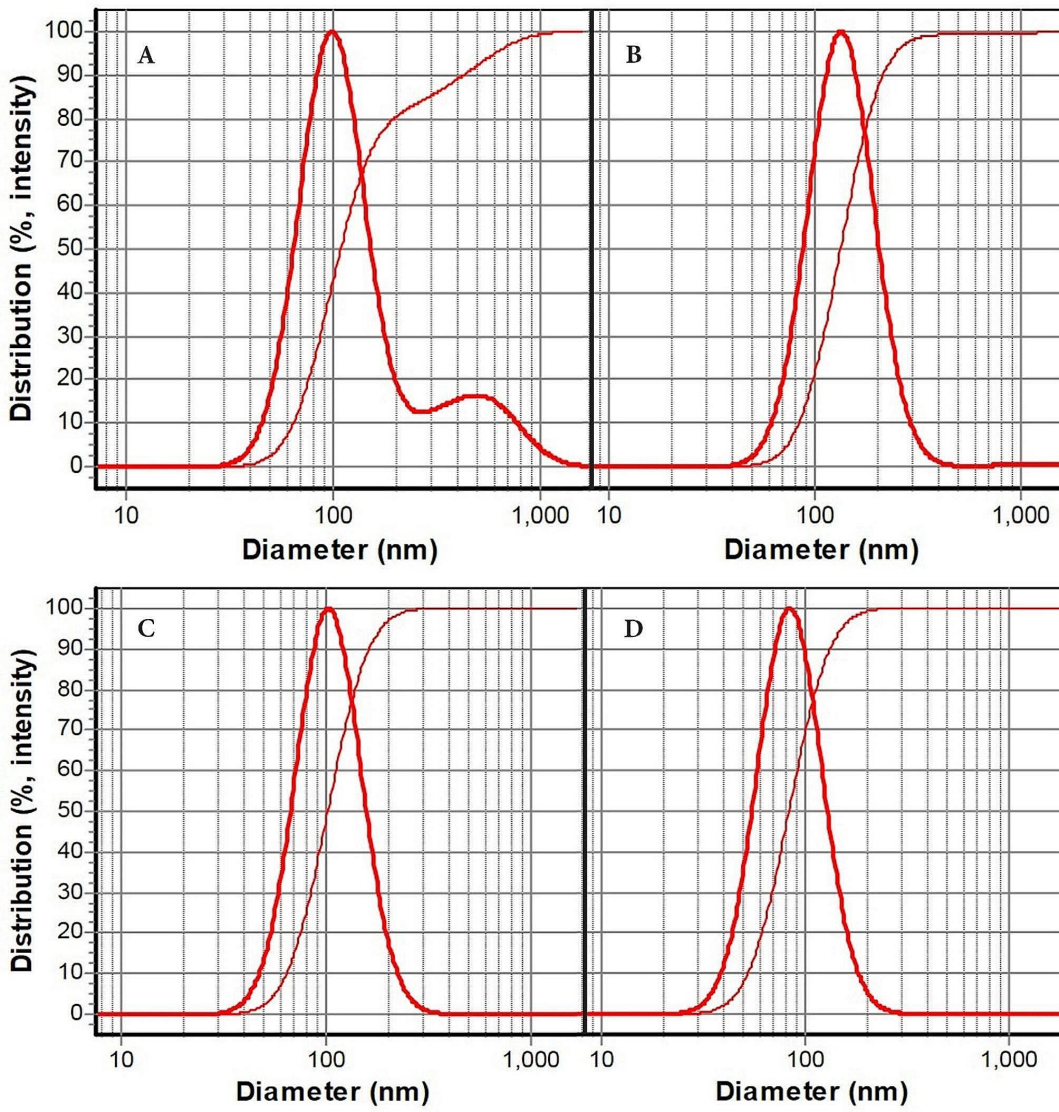
DLS profiles of primary nanoemulsions: **A**: blank nanoemulsion (111 nm), **B**: camphor-thymol nanoemulsion (135 nm), **C**: camphor nanoemulsion (103 nm), and** D**: thymol nanoemulsion (85 nm)

DLS profiles of primary nanoemulsions are shown in Fig. 2A-D. Particle sizes of blank nanoemulsion, camphor-thymol nanoemulsion, camphor nanoemulsion, and thymol nanoemulsion were obtained as 111, 135, 103, and 85 nm, respectively. Their SPAN values were > 1, 0.98, 0.97, and 0.96.

**Fig. 3:**
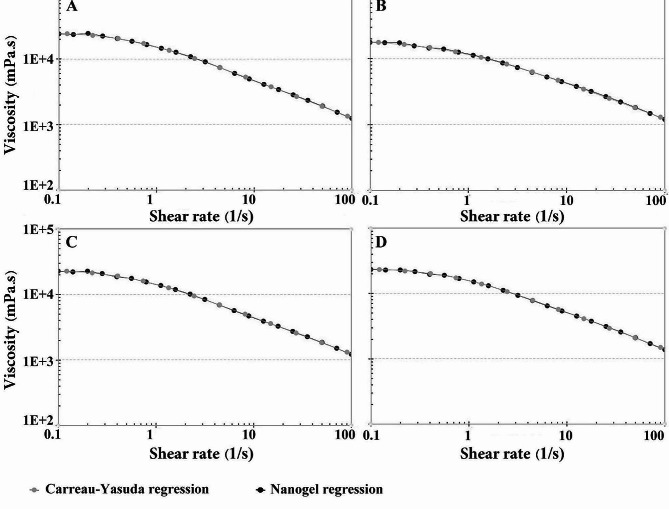
Analyzing the viscosity curves of the nanogels: **A**: blank nanogel (Gel(-C)), **B**: camphor-thymol nanogel (CT-NGEL), **C**: camphor nanogel (C-NGEL), and **D**: thymol nanogel (T-NGEL)

The nanoemulsions were gellified by adding 3.5% w/v of CMC; the viscosity curve of the nanogels, including Gel(-C), CT-NGEL, C-NGEL, and T-NGEL are illustrated in Fig. 3A-D. Their viscosity is fully fitted with a common regression curve for non-Newtonian fluids, i.e., Carreau-Yasuda, where the viscosity decreases by increasing shear rate. Furthermore, all nanogels, Gel(-C), CT-NGEL, C-NGEL, and T-NGEL, remained stable without undergoing any phase separation or dissociation after being stored at ambient temperature and in the refrigerator for six months.

**Fig. 4:**
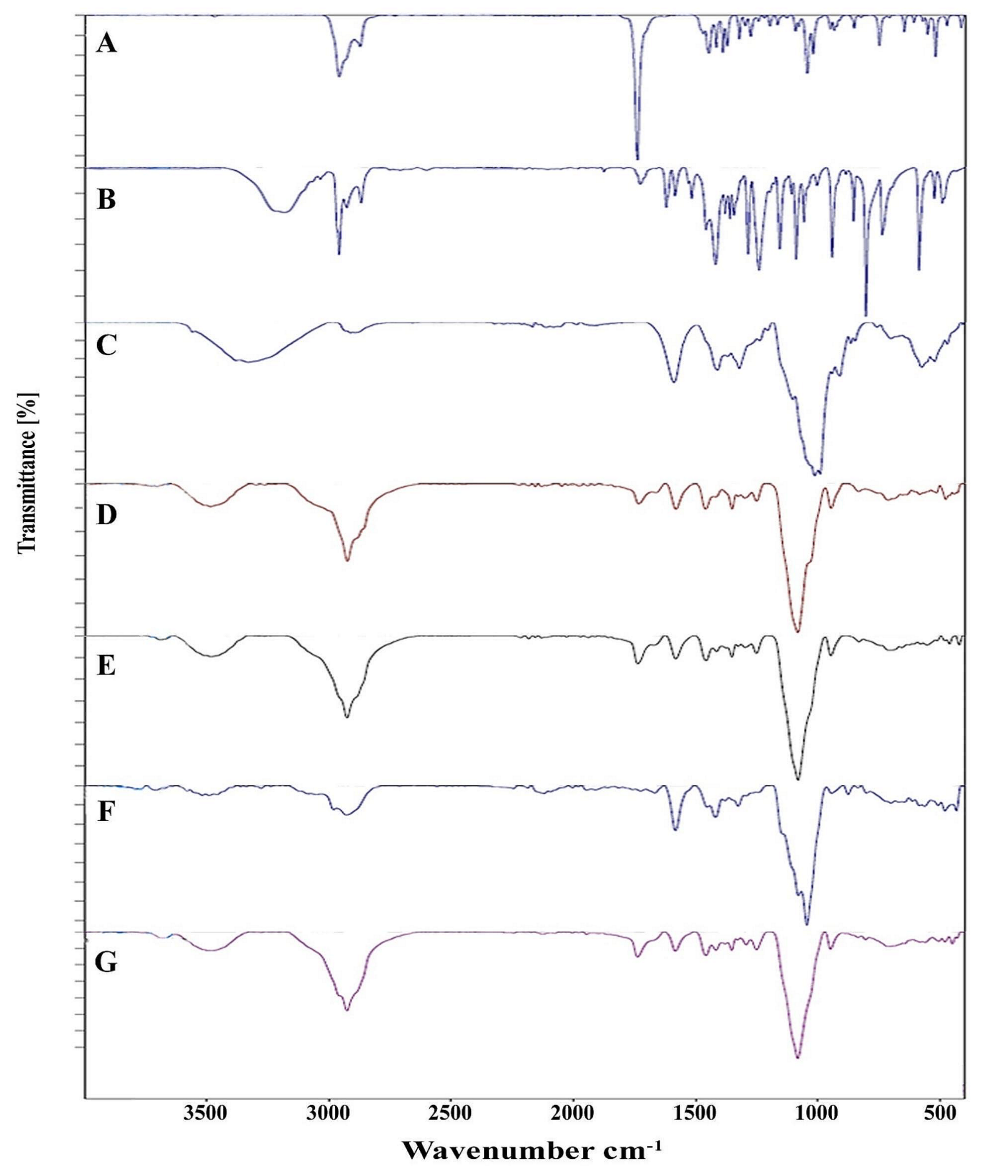
The ATR-FTIR spectra of **A**: camphor, **B**: thymol, **C**: CMC, **D**: balnk nangel (Gel(-C)), **E**: camphor nanogel (C-NGEL), **F**: thymol nanogel (T-NGEL), and **G**:camphor-thymol nanogel (CT-NGEL)

The ATR-FTIR spectrum of camphor (Fig. 4A) exhibited peaks at 2958 and 2872 cm^− 1^, which corresponded to the C-H stretching vibration of hydrocarbon. The presence of the carbonyl group was confirmed by the prominent and intense band observed at 1738 cm^− 1^. The stretching vibration at 1447 cm^− 1^ can be attributed to methylene groups, while the spectra at 1372 cm^− 1^ correspond to methyl groups. The occurrence of C-O stretching was confirmed by a spectrum at 1044 cm^− 1^.

The ATR-FTIR spectrum of thymol (Fig. 4B) exhibited a wide range of spectra extending from 3200 to 3400 cm^− 1^, which can be ascribed to the presence of OH groups resulting from hydrogen bonding. The band observed at 3034 cm^− 1^ corresponds to the = C-H functional group, while the stretching vibrations observed at 2957, 2926, and 2867 cm^− 1^ are associated with the –CH functional group. The absorption peak at 1620 cm^− 1^ is attributed to the vibrational motion of the C = C bond in the aromatic ring of an aromatic compound. The presence of a peak at 1458 cm^− 1^ in the spectrum indicates the presence of an alcohol functional group (C-OH) undergoing bending vibrations during absorption. The stretching vibrations at 124, 1156, and 1057 cm^− 1^ are attributed to the C-O bond and the deformation vibration of C-OH. The absorption at 886 cm^− 1^ correlates with the vibration of C-H bonds in benzene rings. The vibration absorption at 738 cm^− 1^ is assigned to the alkenes.

The ATR-FTIR spectrum of CMC (Fig. 4C) exhibited a wide peak in the 3100–3600 cm^− 1^ range, attributed to the stretching of the hydroxyl group caused by hydrogen bonding. The absorption peak at 1589 cm^− 1^ indicates the presence of the COO group, specifically due to asymmetric stretching. Likewise, the peak at 1411 cm^− 1^ is attributed to the symmetric stretching of the COO group. The spectral pattern observed at approximately 991 cm^− 1^ is associated with the stretching of the C-O bond.

The ATR-FTIR spectrum of Gel(-C) (Fig. 4D) exhibited a broad peak in an area of 3300–3600 cm^− 1^, which can be attributed to the stretching vibration of hydroxyl groups resulting from hydrogen bonding between water and tween 80. The absorption peak observed at around 2924 cm^− 1^ corresponds to the stretching of the C-H bonds, which is prompted by the presence of tween 80 and CMC. The peak observed at 1733 cm^− 1^ relates to the stretching of the C = O bond, which indicates the presence of the carbonyl group in tween 80. The intense and robust peak observed at 1083 cm^− 1^ was ascribed to the stretching of the C-O bond. The COO- band at 1589 cm^− 1^ was observed to move to a lower wave number of 1581 cm^− 1^ in the presence of CMC. This shift confirms the interaction between CMC and tween 80 by intermolecular hydrogen bonding.

The ATR-FTIR spectrum of C-NGEL (Fig. 4E) revealed an extensive peak ranging from 3400 to 3600 cm^− 1^, which can be ascribed to the presence of OH groups resulting from hydrogen bonding between tween 80, water, CMC, and camphor. The spectra at 2923 cm^− 1^ are associated with the stretching of C-H bonds caused by the presence of camphor, tween 80, and CMC. The spectra at 1736 cm^− 1^ verified the existence of C = O stretching, indicating the overlapping carbonyl group in camphor with tween 80. The intense and robust peak observed at 1081 cm^− 1^ is credited to the stretching velocity of the C-O bond. The COO- band at 1589 cm^− 1^, when CMC was present, displayed a noticeable shift towards a lower wave number at 1581 cm^− 1^. This shift confirms the interaction between CMC and tween 80 through intermolecular H-bonding.

The ATR-FTIR spectrum of T-NGEL (Fig. 4F)) exhibits a broad area comprising 3400 to 3600 cm-1, which could be attributed to groups arising from hydrogen bonding. Additionally, the peaks observed at 2979 and 2926 cm^− 1^ are associated with the stretching of C-H bonds, which can be ascribed to the presence of thymol, tween 80, and CMC. The absorption at 1724 and 1666 cm^− 1^ indicates the stretching of the C = O bond, implying the presence of the carbonyl group in thymol and tween 80. The sharp and stable peak at 1079 cm^− 1^ corresponds to the stretching of the C-O bond. The COO- band at 1589 cm^− 1^, when CMC is present, is observed to shift to a lower wave number at 1582 cm^− 1^. This shift validates the link between CMC and tween 80 by intermolecular hydrogen bonding.

The ATR-FTIR spectrum of CT-NGEL (Fig. 4G) revealed a wide band that spans 3400–3700 cm^− 1^, which can be explained by the presence of OH groups resulting from hydrogen bonding between tween 80, water, CMC, thymol, and camphor. The band observed at 2924 cm^− 1^ is attributed to the stretching of C-H bonds, which can be ascribed to the existence of thymol, camphor, tween 80, and CMC. The band observed at a wavenumber of 1736 cm^− 1^ corresponds to the stretching of the C = O bond, which indicates the presence of the carbonyl group in camphor, thymol, and tween 80. The prominent and robust peak observed at 1081 cm^− 1^ belongs to the stretching of the C-O bond. The COO- band at 1589 cm^− 1^, when CMC was present, exhibited an apparent trend towards a lower wave number at 1581 cm^− 1^. This shift confirms an association between CMC and tween 80 through intermolecular H-bonding.

### Antibacterial properties

**Fig. 5:**
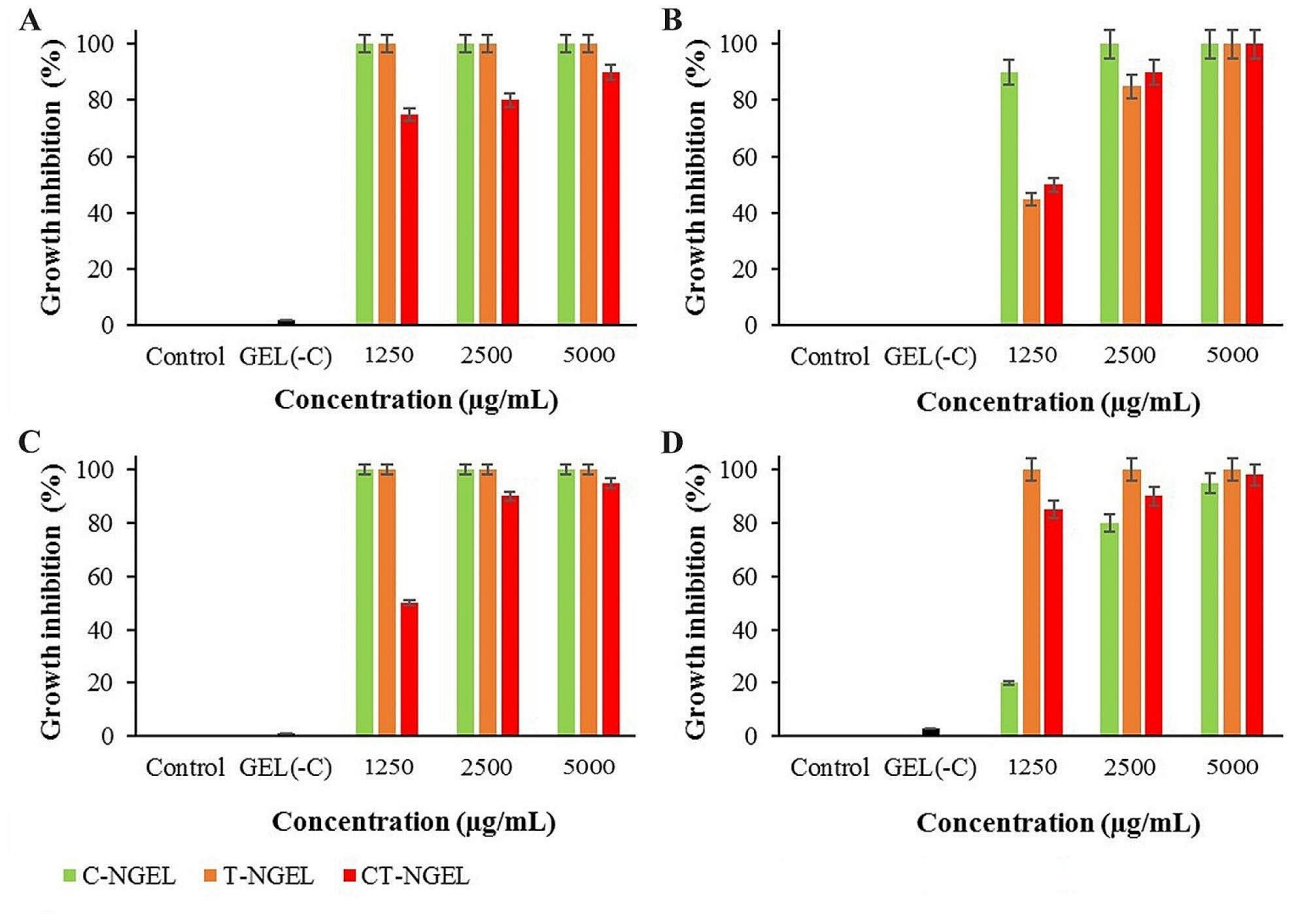
The antibacterial properties of the blank nanogel (Gel(-C)), camphor nanogel (C-NGEL), thymol nanogel (T-NGEL), and camphor-thymol nanogel (CT-NGEL) against **A**: *P. aeruginosa*, **B**: *E. coli*, **C**: *S. aureus*, and **D**: *L. monocytogenes*

Antibacterial effects of Gel(-C), C-NGEL, T-NGEL, and CT-NGEL against *P. aeruginosa*, *E. coli*, *S. aureus*, and *L. monocytogenes* are summarized in Fig. 5A-D. Gel(-C) did not show growth-inhibitory effects on all bacteria. Besides, a positive relationship was observed between the nanogel concentration and their bacterial growth-inhibitory effects. Interestingly, C-NGEL and T-NGEL at 1250, 2500, and 5000 µg/mL showed 100% growth inhabitation on *P. aeruginosa* and *S. aureus*. Besides, C-NGEL (2500 and 5000 µg/mL), T-NGEL (5000 µg/mL), and CT-NGEL (5000 µg/mL) showed 100% growth inhabitation on *E. coli*. Moreover, T-NGEL at 1250, 2500, and 5000 µg/mL showed 100% growth inhabitation on *L. monocytogenes.*

## Discussions

The utilization of herbal medicines (extracts, oil, and essential oil) traces back to ancient civilizations for treating various ailments [32, 33]. In contemporary times, it has garnered substantial attention from researchers and innovators globally owing to its perceived health benefits. Recently, extracts and plant materials have been used to synthesize metal nanoparticle-based antibiotics and develop new antibacterial agents [34, 35]. However, herbal bioactive agents are characterized by low solubility, permeability, and bioavailability. Besides, some bioactive compounds, such as thymol and camphor, possess hydroxyl groups; they are physically and chemically unstable, which reduces their antibacterial activity [36, 37]. Nowadays, it is accepted that nanoformulated herbal ingredients have potential platforms to raise bioavailability, physicochemical properties, high loading capacity, increased solubility, decreased volatility, and target drug delivery [38–40]. Nanoemulsions with straightforward preparation methods, high drug loading capability, and high stability represent pioneering vehicles for encapsulating plant bioactives, enhancing solubility and bioavailability [41, 42]. However, due to its liquid nature, its topical use is challenging. Thus, the present investigation attempted to prepare nanogels incorporating camphor, thymol, and their combination via their primary nanoemulsion, earning the advantages of nanoemulsion and facilitating topical usage simultaneously.

Prior research has extensively documented the diverse array of properties attributed to camphor and thymol, with particular emphasis on their notable antibacterial and antifungal characteristics. Our findings are consonant with this body of literature, which has highlighted camphor’s pivotal role as a predominant constituent in essential oils derived from various botanical sources such as *Lavandula pedunculata, Lavandula dentate* [43], *Lavandula stoechas* [44], *Artemisia annua* [45], *Tanacetum parthenium* [46], *Rosmarinus tournefortii* [47], *Tanacetum parthenium* [48], *Chiliadenus antiatlanticus* [49] exhibiting robust antibacterial efficacy against a wide spectrum of both gram-negative and gram-positive bacteria. Additionally, when utilized as the principal component in cedar leaf essential oil, camphor has demonstrated significant inhibition of *Candida albicans* biofilm formation [50]. Moreover, within the context of *Hedychium spicatum*, camphor has been associated with potent antifungal effects against several phytopathogenic fungi, including *Sclerotinia sclerotiorum, Rhizoctonia solani, Sclerotium rolfsii, and Colletotrichum falcatum* [51]. Despite these remarkable observations, the precise mechanistic underpinnings governing camphor’s antibacterial activity remain partially understood. Current evidence suggests that its antimicrobial effects may be attributed, in part, to the reduction of the pH gradient, destabilization of the cell membrane’s double-layer structure, and interaction with membrane-bound enzymes and proteins [43, 52, 53]. Besides, camphor exerts an analgesic action when used topically, producing a warm sensation. It excites and desensitizes sensory nerves by activating heat-sensitive TRP vanilloid subtype 1 (TRPV1) and TRPV3 receptors [54, 55]. This intricate interplay underscores the multifaceted nature of camphor’s bioactivity and the need for further elucidation through comprehensive mechanistic studies.

From the literature, thymol has demonstrated remarkable effectiveness against numerous gram-positive bacterial strains, including but not limited to *Staphylococcus aureus* [56, 57], *Staphylococcus epidermidis, Streptococcus mutans* and *Bacillus subtilis* [58], *Bacillus cereus* [59, 60], as well as an extensive roster of gram-negative bacteria such as *Enterobacter sakazakii* [61], *Escherichia coli* [57, 59], *Pseudomonas aeruginosa* [57, 59], *Aeromonas hydrophila* [62], *Salmonella Infantis* [60], *Salmonella typhimurium* [63], *Salmonella paratyphi* [63], *Shigella flexneri* [63]. Moreover, comparable to camphor, thymol has been observed to exhibit profound antifungal properties, effectively combatting various species within the *Candida* species [64–66], *Trichophyton* species [67], and *Cryptococcus neoformans* [68, 69]. The mechanisms underpinning thymol’s antibacterial prowess are multifaceted, encompassing both membrane-related and intracellular actions. Its lipophilic nature facilitates its interaction with bacterial lipid membranes, disrupting membrane pumps and enzymes crucial for bacterial survival [21, 70]. Additionally, thymol’s ability to bind with large intracellular macromolecules, such as DNA, induces structural alterations within bacterial cells, ultimately culminating in bacterial death [71, 72]. This intricate interplay between thymol and microbial targets underscores its potential as a versatile antimicrobial agent, warranting further investigation and exploitation in the ongoing quest for novel therapeutic interventions against infectious diseases.

Our study bridges the gap between traditional herbal remedies and modern nanoformulation techniques. The findings unveil a potent antimicrobial activity whereby these compounds effectively curbed the growth of *P. aeruginosa, S. aureus, L. monocytogenes*, and *E. coli* at concentrations equal to or below 2500 µg/mL. Unlike prior studies where camphor and thymol were examined either as predominant constituents of essential oils or in their pure forms, our approach involved their nanogel dosage form as a practical form. Recently, nanogels containing essential oils or plant compounds have been widely used in animal models. For instance, nanogels containing cinnamon essential oil and eugenol showed promising effects in wound healing [73, 74]. It seems that the achievements of such studies have reached enough maturity to measure their effectiveness in clinical trials, although their safety considerations must be observed.

## Conclusion

In this study, an attempt was made to develop nanogels of camphor, thymol, and their mix. The efficiency of camphor and thymol nanogels was higher than their mixture. Both C-NGEL and T-NGEL showed 100% inhibitory effects on *P. aeruginosa* and *S. aureus*. Besides, C-NGEL and T-NGEL showed 100% growth inhibitory effects on *L. monocytogenes* and *E. coli*, respectively. Considering the straightforward preparation method and high efficacy, they could be introduced as candidates for other pathogens and in vivo studies.

## Data Availability

All data are available by reasonable request from the corresponding author.
